# Establishing the First Genetic Variant Registry for Breast and Ovarian Cancer in Colombia: Insights and Implications

**DOI:** 10.3390/diseases13070222

**Published:** 2025-07-18

**Authors:** Robert de Deugd, Julián Camilo Riano, Esther de Vries, Andrés F. Cardona, July Rodriguez, Ana Fidalgo-Zapata, Yesid Sanchez, Santiago Sanchez, Justo Olaya, Daniel de Leon, Carlos Andrés Ossa, Humberto Reynales, Paula Quintero, Elizabeth Vargas, Ute Hamann, Diana Torres

**Affiliations:** 1 FilaTech Technology GmbH, 53424 Remagen, Germany; r.de.deugd@filatech.de; 2 National Cancer Institute, Bogotá 111511, Colombia; jcriano@cancer.gov.co; 3 Department of Clinical Epidemiology and Biostatistics, Pontificia Universidad Javeriana, Bogota 110311, Colombia; estherdevries@javeriana.edu.co; 4 Foundation for Clinical and Applied Cancer Research (FICMAC), Bogotá 110111, Colombia; acardona@fctic.org (A.F.C.); laboratorio@ficmac.org (J.R.); 5 Surgery-Mastology, Cinica de Occidente, Cali 760001, Colombia; ana.fidalgo.asi@quironsalud.com; 6 Gynecologic Oncologist, Universidad de Tolima, Tolima 730001, Colombia; yesid.sanchez@yahoo.com; 7 Epidemiologist, Oncodiagnóstico SAS, Tolima 730001, Colombia; sa.sanchez@javeriana.edu.co; 8 Surgery-Mastology, Unidad Oncologica Surcolombiana, Neiva 410001, Colombia; jolayaramirez@yahoo.com; 9 Surgery-Mastology, Clinica Central de Quindio, Armenia 630001, Colombia; danieldeleono@hotmail.com; 10 Surgery-Mastology, Centro de Excelencia en Mama de Antioquia, Medellin 050035, Colombia; info@drandresossa.com; 11 Center for Medical Care and Research, Center for Medical Care and Research, Cundinamarca 250001, Colombia; humberto.reynales@caimed.com; 12 Independent Researcher, Bogotá 110111, Colombia; quinteropaula@gmail.com; 13 Institute of Human Genetics, Pontificia Universidad Javeriana, Bogotá 110311, Colombia; elizabeth.vargas@javeriana.edu.co; 14 German Cancer Research Center (DKFZ), Molecular Genetics of Breast Cancer, 69120 Heidelberg, Germany; ute.hamann@alumni.uni-heidelberg.de

**Keywords:** cancer-associated genes, genetic variants, breast cancer, ovarian cancer, cancer registry, precision medicine

## Abstract

Background: Genetic insights from diverse populations are key to advancing cancer detection, treatment, and prevention. Unlike other Latin American countries, Colombia lacks a centralized registry for germline and somatic mutations in breast and ovarian cancer. This study describes the country’s first national variant registry, and the occurrence of recurrent mutations and potential founder effects in Colombia. Methods: To address this gap, we implemented the first capturing protocol using the REDCap system. In a group of 213 breast and/or ovarian cancer patients harboring genetic mutations, we collected genetic, clinical, and demographic data from 13 regional centers across Colombia. Statistical analyses assessed variant distribution and patient demographics. Results: Among 229 identified variants (105 germline, 124 somatic), most were classified as pathogenic or likely pathogenic (72.4% germline, 87% somatic). *BRCA1* and *BRCA2* accounted for the majority of recurrent mutations. Germline recurrent variants (seen >3 times) were recorded for *BRCA1* (77.7%; 21/27) and *BRCA2* (22.3%; 6/27). Similarly, recurrent somatic variants were identified for *BRCA1* (82.6%; 38/46) and *BRCA2* (17.4%; 8/46). Notably, four recurrent variants were previously reported as founder mutations: *BRCA1* c.1674del (14.3% germline and 23.7% somatic), *BRCA1* c.3331_3334del (33.3% germline and 52.6% somatic), *BRCA1* c.5123C>A (52.4% germline and 23.7% somatic), and *BRCA2* c.2808_2811del (50% germline and 50% somatic). Most cases originated from the Andean region, highlighting regional disparities. Conclusions: This registry offers the first overview of genetic variants in Colombian breast and ovarian cancer patients. Recurrent and region-specific mutations highlight the need for population-focused data to guide targeted screening and personalized care strategies.

## 1. Introduction

Breast cancer (BC) remains a leading global public health concern, ranking as the most commonly diagnosed cancer among women and the second most prevalent cancer overall. Ovarian cancer (OC) is the most lethal gynecologic malignancy, partially due to its asymptomatic presentation and frequent diagnosis at late stages. Although the global incidence of both BC and OC varies—typically higher in high-income countries—these diseases continue to impose significant health and economic burdens across all regions, including Latin America [1].

From a genetic standpoint, cancer is driven by both inherited (germline) and acquired (somatic) mutations. Germline pathogenic or likely-pathogenic (P/LP) mutations are present in all cells of a person and can increase cancer susceptibility. Somatic mutations occur in specific tissues and can be principal catalysts for cancer initiation and progression [2]. 

Conducting hereditary cancer risk assessments is at the forefront of identifying individuals and families potentially at an elevated risk of developing specific cancer types. The detection of a hereditary (germline) pathogenic/likely-pathogenic (P/LP) variant can profoundly impact decisions related to pharmacologic treatments, specialized prevention programs, and cascade testing within families [3].

Important germline mutations related to breast and ovarian cancer such as *BRCA1* and *BRCA2* (*BRCA1/2*) have been identified [4], and other high- and moderate-risk genes have been found to be associated with increased risk for these cancers [5].

Genetic testing for germline mutations—particularly for the *BRCA1* and *BRCA2* genes—is a key component of hereditary cancer risk assessment and its results can influence treatment choices, preventive interventions, and family-based cascade testing. In addition to these germline mutations, somatic mutations are increasingly recognized as actionable therapeutic targets. For example, poly(ADP-ribose) polymerase (PARP) inhibitors such as Olaparib and Talazoparib have demonstrated efficacy in patients with BRCA-mutated tumors [6]. Early detection of these mutations plays a pivotal role in the effective management of cancer, significantly improving patient outcomes.

Although genetic testing has been integrated into routine oncology care in Colombia [7], the country lacks a dedicated registry for cataloging breast and ovarian cancer-related gene variants—whether germline or somatic. This absence limits our ability to understand the mutational spectrum within our population and impedes the development of tailored public health policies and clinical guidelines [8].

To address this gap, we established Colombia’s first national registry of genetic variants associated with breast and ovarian cancers. This structured resource compiles germline and somatic P/LP variants and variants of uncertain (or unknown) significance (VUSs), with a focus on *BRCA1* and *BRCA2* and an expanded panel of other relevant high- and moderate-risk genes existing in our population. These data allowed us to analyze data from diverse regions across Colombia, and in that way contribute to the characterization of the local mutational landscape and generate population-specific insights. This dataset has the potential to equip medical professionals and public health institutions with more precise, population-specific insights to inform strategies aimed at controlling these high-burden malignancies.

Unlike international databases such as ClinVar, BRCA Exchange, or LOVD—which focus on variant classification and cross-institutional harmonization—the primary purpose of our registry is as a national reference tool to document the observed mutations in our population. Its primary value lies in providing an epidemiological repository to inform national public health planning, supporting national research efforts. In addition, it can contribute to a more inclusive representation of Latin American populations in global genomic databases. The objective of this paper is to present the design, development, and initial findings of Colombia’s first breast and ovarian cancer variant registry, highlighting its structure, scope, and potential contributions to research and public health policy.

## 2. Materials and Methods

Data collection for this study involved thirteen collecting centers (CCs), defined as Colombian genetic counseling units recognized as reference institutions that provided genetic reports for 213 patients diagnosed with breast cancer (BC), ovarian cancer (OC), or both. A total of 229 gene variants were registered. The cases included in this registry were retrospectively selected based on the availability of genetic testing results in patients who received care at one of the 13 participating centers and had a confirmed diagnosis of BC, OC, or both. All patients provided informed consent for the use of their clinical and genetic data. The case selection period spanned from January 2013 to December 2022, covering the ten years prior to the final data consolidation in 2023.

**Figure 1:**
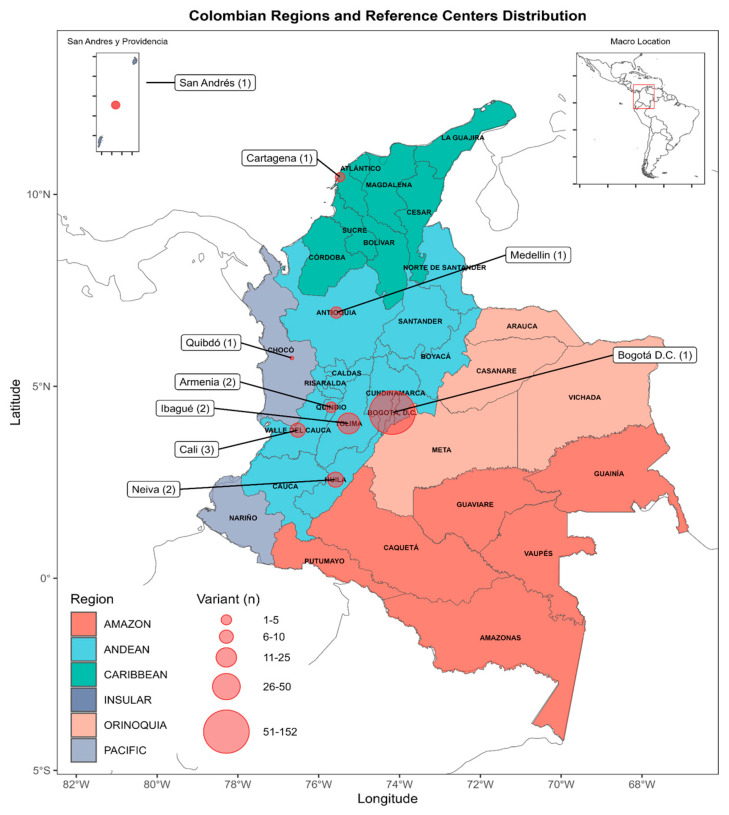
Geographic distribution of the 13 Collecting Centers (CCs) contributing to the Colombian breast and ovarian cancer genetic variant registry. The map displays Colombia’s six main regions—Andean, Caribbean, Pacific, Orinoquia, Amazon, and Insular—and indicates the location of each reference center. Each bubble is labeled with the corresponding city name and the number of participating centers. The size of each bubble is proportional to the number of genetic variants reported by each location, visually reflecting regional contributions to the 229 registered variants.

Among the 13 contributing centers, the majority—six (46.1%)—were located in the Andean region, highlighting this region’s predominant representation in the dataset. The Pacific and Caribbean regions were each represented by two centers (15.3%), while the Amazon, Orinoquia, and Insular regions each had one contributing center. This ensured inclusion of data from geographically diverse areas, albeit with more limited representation in remote regions. Overall, the participating centers were distributed across nine departments spanning Colombia’s six distinct geographical regions (Figure 1).

Genetic testing was not centralized; molecular analyses were performed in accredited laboratories using validated sequencing platforms and bioinformatics pipelines. Variant data were extracted directly from clinical reports, without reprocessing or reanalysis of raw sequencing data. As such, no harmonization across platforms was applied. All testing laboratories followed internationally accepted variant classification standards, including ACMG/AMP guidelines.

Variants were documented as originally reported and used in clinical decision making. No independent reclassification was performed, and reclassification over time was not assessed. Although some laboratories may update variant interpretations—especially for VUS—this registry did not track such changes. The objective was to capture germline and somatic variants, including VUSs, based on the information available at the time of diagnosis.

**Figure 2:**
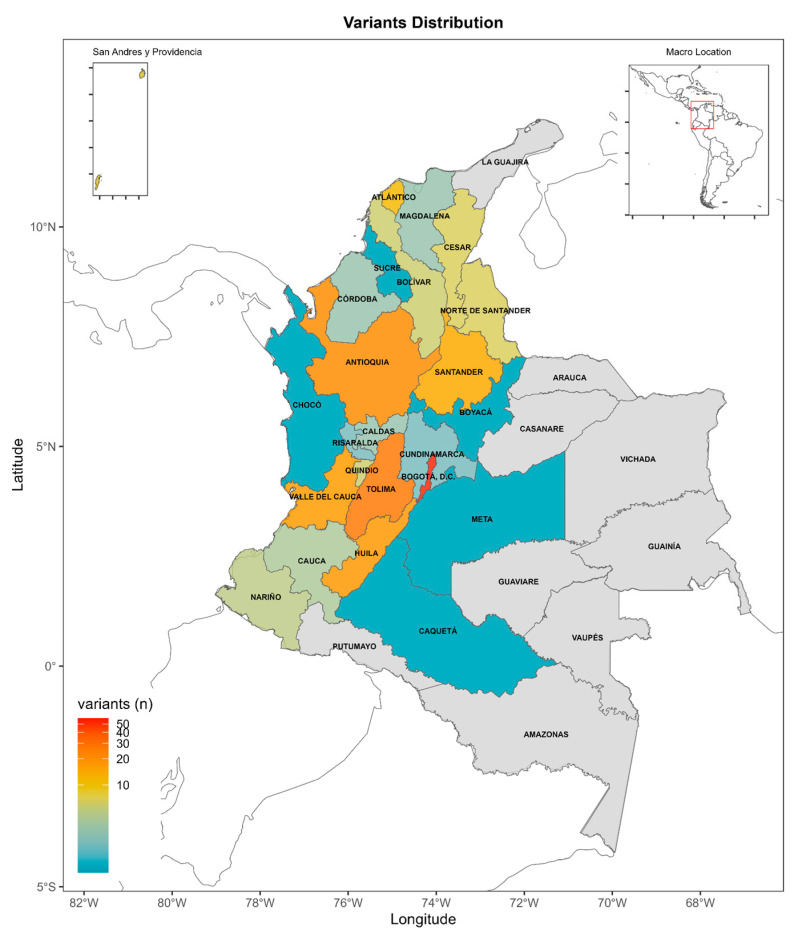
Geographic distribution of the 229 registered genetic variants—including 105 germline and 124 somatic mutations—across Colombia’s six main regions: Andean, Caribbean, Pacific, Orinoquia, Amazon, and Insular. This map illustrates the regional spread and frequency of reported variants, offering insights into geographic patterns and potential regional clustering of specific mutations. The visualization emphasizes the predominance of reported variants in the Andean region, while also drawing attention to the limited data from remote and underserved areas.

The contribution of variants by region was as follows: Andean (71.6%; 164/229), Caribbean (14.4%; 33/229), Pacific (12%; 28/229), and Insular (1%; 2/229). Only one entry was observed in each of the Amazonia and Orinoquia regions, each contributing 0.5% (1/229) to the overall dataset. Participant identification and baseline information collection occurred at their respective centers, ensuring a representative and diverse sampling across the country (Figure 2).

A total of 213 patients were included in the study, comprising 93 (44%) diagnosed with BC, 114 (53%) with OC, and 6 (3%) with BOC, all harboring genetic mutations and providing informed consent. Utilizing state-of-the-art technology, specifically targeted gene panel analysis employing next-generation sequencing (NGS) molecular genetic testing was conducted to ascertain their inherited susceptibility to these malignancies. Variant classification followed the American College of Medical Genetics and Genomics (ACMG) and the Association for Molecular Pathology (AMP) guidelines for ACADVL variant interpretation, as outlined by Flowers et al. [9]. Variants were categorized as pathogenic/likely-pathogenic (P/LP) or variants of uncertain significance (VUSs).

Comprehensive data spanning clinical, demographic, and genotyping information was meticulously gathered from all participants. This information was procured via a meticulously designed and standardized questionnaire administered and completed by the attending physicians of the patients. The study aimed to provide a nuanced and thorough comprehension of the genetic variants associated with breast and ovarian cancers, thereby elucidating potential risk factors and enhancing the precision of clinical decision making.

The research protocol received approval from the Ethics Committee of the Pontificia Universidad Javeriana, Bogotá, Colombia (06/2021). We utilized the institutional REDCap (Research Electronic Data Capture) system for data capture, storage, and management (Edition N°: 1365).

### 2.1. Data Processing

The data preparation process included handling incomplete genetic reports provided by the collecting centers. This involved excluding entries where genetic information was ambiguous, unreadable, or incomplete. In cases where the genetic reports were unclear or contained anomalies that could compromise the integrity of our analysis, they were systematically removed from the dataset. The data cleaning and wrangling process was performed using the R programming language, utilizing the packages tidyverse, dplyr, ggplot2, and GenVisR. We streamlined the dataset by eliminating redundancies, reformatting inconsistent data entries, and addressing missing values.

### 2.2. Statistical Analysis

Descriptive statistics were used to summarize clinical and demographic variables. Continuous covariates were exclusively described using medians and interquartile ranges (IQR), as most variables did not follow a normal distribution. The Kruskal–Wallis rank-sum test was applied to compare continuous and ordinal variables across diagnostic groups, age categories, and geographic regions. For associations between categorical variables, Fisher’s exact test was used due to small sample sizes and its robustness in 2 × 2 contingency tables.

## 3. Results

As of 2022, the registry included data on 229 genetic variants identified in 213 individuals diagnosed with breast and/or ovarian cancer over the past decade. Among these patients, 93 (44%) were diagnosed exclusively with BC, 114 (53%) with OC alone, and 6 (3%) with both breast and ovarian cancer (BOC). The median age of onset for BC was 45 years (range: 23–76 years), and for OC it was 61 years (range: 32–82 years) (Table 1).

Seventy-seven out of the 93 BC cases (83%) were invasive, while 4 cases (4%) were in situ, and for 12 patients there was no information on the invasiveness of the cancer. Among the invasive BC cases, most were of the triple-negative molecular subtype (49/93 patients (53%)), followed by Luminal B (16%), Luminal A (15%), and Her-2-Enriched (9.6%) subtypes. In patients diagnosed with OC, the high-grade serous histotype was the most common, found in 87 out of 114 cases (76%). The ‘other’ category represented 14%, followed by the endometrioid (4.4%), low-grade serous (2.9%), clear cell (1.8%), and mucinous (0.9%) subtypes (Table 2).

The analysis revealed a total of 229 distinct variants. Of these, 105 were germline variants, with 76 (72.4%) classified as pathogenic/likely pathogenic (P/LP) and 29 (27.6%) as VUSs. The remaining 124 variants were somatic, including 108 (87%) classified as P/LP and 16 (13%) as VUSs (Tables S1 and S2).

Notably, 16 patients (7.5%) harbored two variants. In most of these cases, the combination involved a P/LP variant alongside a VUS, or the same variant was identified in both peripheral blood and formalin-fixed paraffin-embedded (FFPE) tissue. Among the multilocus P/LP cases, one BOC patient carried two germline pathogenic variants in different genes—*BRCA2* and *POLD1*. Additionally, three OC patients were found to carry two somatic P/LP variants: one had mutations in different genes (*BRCA1* and *BRCA2*) and the remaining two patients carried two somatic mutations within *BRCA1*.

### 3.1. Registration of Variants by Region

Our analysis of variant distribution across six Colombian regions revealed a marked predominance of reports from the Andean region, accounting for 164 out of 229 total variants (71.6%). Among these Andean variants, 45 germline P/LP variants (68.1%) were identified in key susceptibility genes including *ATM*, *BRCA1*, *BRCA2*, *BRIP1*, *MLH1*, *MUTYH*, *PMS2*, *POLD1*, and *RAD51C*. Additionally, 21 variants (31.9%) were classified as VUSs. The Andean region also contributed the majority of somatic findings, with 86 (87.7%) P/LP somatic variants in *BRCA1 * and *BRCA2*.

The Caribbean region contributed 33 variants (14.4%), including 17 (77.3%) germline P/LP variants, predominantly in *BRCA1* and *BRCA2*, and 5 (22.7%) VUSs. Somatic variants from this region included 10 P/LP variants (91%) in *BRCA1*/2, and 1 VUS (9%).

In the Pacific region, 28 (12%) variants were recorded. Germline P/LP variants made up 12 of these (86%), associated with *ATM*, *BRCA1*, *BRCA2*, *MUTYH*, and *PTEN*, 2 variants were (14%) VUSs. Somatic variants showed a similar trend, with 11 (78.5%) identified as P/LP, again mostly involving *BRCA1*/2, and 3 (21.5%) identified as VUSs.

Very few cases were reported from the Insular (2/229; 1%), Orinoco (1/229; 0.5%), and Amazon (1/229; 0.5%) regions. Each of these regions contributed one or two variant entries.

### 3.2. Recorded Germline Variants

**Figure 3:**
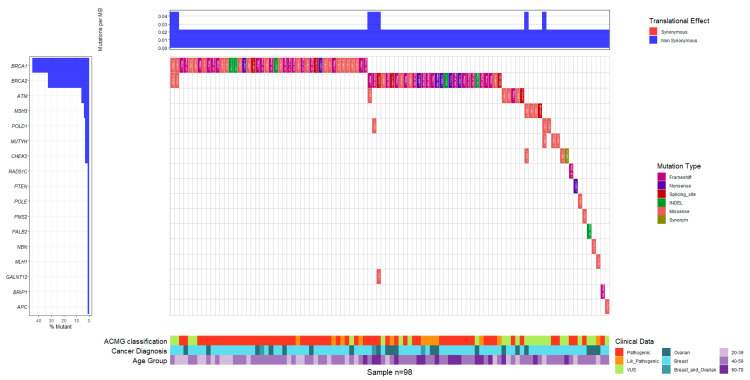
Waterfall plot displaying the distribution and frequency of germline variants identified across breast cancer (BC), ovarian cancer (OC), and combined BC/OC cases. Each bar represents an individual case, categorized by cancer type and the specific gene in which the variant was found. The plot includes both pathogenic/likely pathogenic (P/LP) variants and variants of uncertain significance (VUS), classified according to ACMG (American College of Medical Genetics and Genomics) guidelines. INDELs (insertions and deletions) are represented alongside single nucleotide variants (SNVs), offering a comprehensive overview of the germline mutational spectrum. This visualization highlights the predominance of *BRCA1* and *BRCA2* mutations and underscores the relevance of incorporating structured genetic screening in high-risk populations.

A total of 105 germline variants (45.9%) were reported, with 76 (72.4%) being classified as P/LP and 29 (27.6%) as VUSs. Among these variants, 79 (75%) were in BC patients, 22 (21%) in OC patients, and 4 (4%) in patients with BOC. The most frequently represented genes were *BRCA1*, with 40 occurrences (52.6%), and *BRCA2*, with 26 occurrences (34.2%). In contrast, only one or two variants were reported in the remaining genes, including *ATM*, *MUTYH*, *BRIP1*, *MLH1*, *PMS2*, *POLD1*, *PTEN*, and *RAD51C* (Figure 3).

Among the 76 germline P/LP variants identified, the *BRCA1* c.5123C>A emerged as the most frequent, accounting for 11 of the 21 (52.4%) recurrent *BRCA1* variants. This variant demonstrated a regional distribution skewed towards higher prevalence in the Caribbean area, where it was detected in six cases (54.6%), followed by the Andean region with four cases (36.4%) and a single occurrence in the Orinoquia region (9%). The second most prevalent pathogenic variant was *BRCA1* c.3331_3334del, observed in seven cases (33.3%). This variant was predominantly found in the Andean region (five cases; 71.4%), with one case each in the Caribbean and in the Pacific regions (14.3% each). Other recurrent variants included *BRCA1* c.1674del and *BRCA2* c.2808_2811del and c.4889C>G, with frequencies ranging from 14.3% to 50%. These variants were observed in the Andean, Caribbean, and Pacific regions (Table 3).

### 3.3. Recorded Somatic Variants

**Figure 4:**
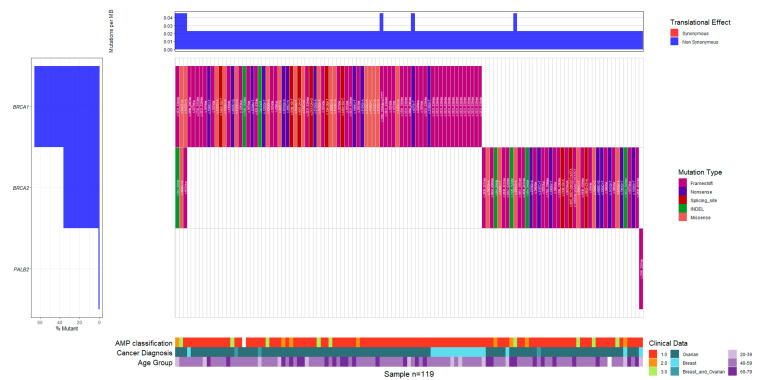
Waterfall plot illustrating the distribution and frequency of somatic variants identified in patients diagnosed with breast cancer (BC), ovarian cancer (OC), or both. Each bar represents an individual case, detailing the specific gene affected and the variant type. The plot includes pathogenic/likely pathogenic (P/LP) variants and variants of uncertain significance (VUS), classified based on AMP (Association for Molecular Pathology) guidelines. Both single nucleotide variants (SNVs) and INDELs (insertions and deletions) are depicted, providing a comprehensive overview of the somatic mutational landscape. The predominance of *BRCA1* and *BRCA2* alterations is evident, reinforcing their clinical relevance in somatic tumor profiling and targeted treatment approaches.

A total of 124 somatic variants (54.1%) were reported, of which 108 (87%) were classified as P/LP and 16 (13%) as VUSs. Among these 124 somatic variants, 18 (15%) were identified in BC patients, 103 (83%) in OC patients, and 3 (2%) in patients with both cancers. The majority of these somatic variants were located in the *BRCA1* (73/108; 67.6%) and *BRCA2* (34/108; 31.4%) genes. Only one somatic P/LP variant outside of the *BRCA1*/2 genes was detected—*PALB2* c.2288_2291del (Figure 4).

Among the 108 somatic P/LP variants identified, *BRCA1* c.3331_3334del was the most frequently observed, accounting for 20 out of 38 variants (52.6%) observed in this gene. The majority of these cases (95%) were recorded in the Andean region, with a single report from the Amazon region. The next most common *BRCA1* pathogenic variants were c.5123C>A and c.1674del, each identified in nine cases (23.7% each), and distributed across the Andean, Caribbean, and Pacific regions. In the *BRCA2* gene, the most recurrent pathogenic variants were c.2808_2811del and c.3860del, each observed in four out of the eight *BRCA2* cases (50%, Table 3).

### 3.4. Recorded Germline Pathogenic/Likely Pathogenic Variants and Age of Breast/Ovarian Diagnosis

Among the 93 BC patients (43.7% of the whole group), 59 (63.4%) carried germline P/LP variants, predominantly in the *BRCA1* (56%) and *BRCA2* (30.5%) genes. The *BRCA1* c.5123C>A variant was the most frequently identified one, accounting for 11 out of 33 *BRCA1* variants (33.3%). In *BRCA2*, the most commonly identified variants were c.2808_2811del and c.4889C>G, each observed in 3 out of 18 cases (16.7% each). Additional P/LP variants were detected in the *ATM*, *BRIP1*, *MUTYH*, *PMS2*, *PTEN*, and *RAD51C * genes, with presence in one or two variants each.

An age-stratified analysis showed that the 40–59 years group comprised the majority of BC cases (58/93; 62%), followed by the 20–39 years group (26/93; 28%) and the 60–79 years group (9/93; 10%).

A similar pattern was observed among the 114 OC cases (53.5% of the total group), with *BRCA1* and *BRCA2* being the most prevalent genes. Of these, 22 patients (19.3%) carried germline P/LP variants, predominantly in the *BRCA1* (46.2%) and *BRCA2* (46.2%) genes. One additional variant was documented in the *MLH1* gene. The *BRCA1* c.3331_3334del variant emerged as the most frequently recorded (4/6; 66.7%) in the *BRCA1* gene, while no recurrent variants were observed in *BRCA2*.

Age at diagnosis followed a similar distribution as breast cancer, with 66% (75/114) diagnosed between 40 and 59 years, followed by 26% (30/114) in the 60–79 years group and 7% (8/114) in the 20–39 age group. Only one patient was diagnosed over the age of 80.

The small group of patients who were diagnosed with both breast and ovarian cancer had germline P/LP variants in *BRCA1* (25%), *BRCA2* (50%), and *POLD1* (25%). Within this BOC group, BC diagnosis occurred in one patient (17%) in the 20–39 age group, two (33%) were aged 40–59, two (33%) aged 60–79, and one (17%) was in the 80–99 age group when diagnosed with the BC. For OC diagnosis in this same group, three patients (50%) were aged 40–59 and the other three individuals (50%) were between 60 and 79 years old when diagnosed with OC.

### 3.5. Recorded Germline and Somatic Pathogenic/Likely Pathogenic Variants in Breast and Ovarian Histopathological Types

Among BC patients, the molecular histopathological subtypes included Her-2-enriched (9/93; 9.6%), Luminal A (14/93; 15%), Luminal B (15/93; 16%), and triple negative (49/93; 53%). Triple-negative breast cancer was the most prevalent subtype and was associated with the recurrent *BRCA1* germline variants c.3331_3334del and c.5123C>A. Additionally, single P/LP germline variants in the *PTEN* and *RAD51C* genes, as well as a somatic variant in *PALB2*, were associated with triple-negative breast cancer. The Luminal A and Luminal B subtypes, the second most common in this database, were primarily associated with germline and/or somatic P/LP variants in *BRCA1*/2, including the aforementioned recurrent ones. Unique variants in the *ATM*, *BRIP1*, and *MUTYH* genes were also observed in these subtypes, suggesting additional genetic heterogeneity.

Among OC patients, the histological subtypes observed included clear-cell carcinoma (2/114; 1.8%), endometrioid (5/114; 4.4%), mucinous (1/114; 0.9%), other subtypes (16/114; 14%), low-grade serous (3/114; 2.9%), and high-grade serous carcinomas (87/114; 76%). High-grade serous carcinomas were by far the most prevalent, and among this group, recurrent variants were reported in both the *BRCA1* (c.1674del, c.3331_3334del, and c.5123C>A) and *BRCA2* (c.3860del) genes, detected at the germline and/or somatic level. Additionally, a single germline variant in the *POLD1* gene was found to be associated with this histotype.

## 4. Discussion

Advances in sequencing technology have significantly enhanced our understanding of hereditary and somatic cancers. This study aimed to establish a population-based cancer mutation gene registry in Colombia, focusing primarily on *BRCA1/2* variants and other genes associated with breast and ovarian cancer. Our findings confirm that *BRCA1* and *BRCA2* have a central role in hereditary breast and ovarian cancer syndromes in the Colombian population, with these genes accounting for 87% of P/LP germline variants and 99% of somatic P/LP variants. *BRCA1* variants were the most frequent at both the germline (52.6%) and somatic (67.6%) levels, followed by *BRCA2* variants (34.2% germline; 31.5% somatic).

Several recurrent germline P/LP variants—such as *BRCA1* c.5123C>A, *BRCA1* c.3331_3334del, and *BRCA2* c.2808_2811del—have been reported as potential founder mutations in Colombian families [10]. The identification of additional variants such as *BRCA1* c.1674del and *BRCA2* c.4889C>G, along with three new large genomic rearrangements (LGRs) supports the need for a haplotype analysis to investigate possible founder effects [11]. The similar occurrence between germline and somatic P/LP variants distributions suggests that there are potential population-specific mutational hotspots.

The identification of patients with multi-locus inherited neoplasia allele syndrome (MINAS), such as the individual with co-occurring *BRCA1* and *POLD1* variants, illustrates the complexity of hereditary cancer syndromes and the need for tailored clinical surveillance and care [12]. Age-related breast and ovarian patterns in our data align with international observations: BC peaks in between 50 and 69 years, and ovarian cancer (OC) incidence increases after 50 [13]. Early screening is recommended for younger, higher-risk women, especially those with family histories or genetic mutations [14].

Our study also reveals that patients in Colombia are often diagnosed at invasive stages of cancer, with the invasive type accounting for 83% of BC cases and 76% of OC cases. In BC, triple-negative breast cancer (TNBC) is most common, emphasizing the need for regular screening and risk-reducing strategies for women with *BRCA1* mutations [15]. Targeted therapies, such as PARP inhibitors, show promise for treating *BRCA1/2*-mutated cancers, including TNBC and high-grade serous ovarian cancer [16].

Our findings are consistent with global patterns in BRCA-related cancer risk but also underscore the under-representation of Latin American populations in international variant databases such as ClinVar, BRCA Exchange, and LOVD. Several variants frequently observed in our registry have limited or no representation in these databases, highlighting the critical importance of national and regional data to improve variant interpretation and reduce health disparities in precision oncology.

However, the registry’s scope and generalizability are limited by several factors. First, the data are disproportionately derived from Colombia’s Andean region, reflecting regional disparities in healthcare infrastructure and access to genetic services [17]. Although regions like Insular, Orinoco and Amazon regions have very low population density, the reporting of just one or two cases per region clearly illustrates a lack of resources and reporting in such areas. This geographic concentration introduces sampling bias and limits the representativeness of variant frequencies nationwide. Second, incomplete clinical information—such as lack of tumor staging or treatment data—hampers more robust genotype–phenotype correlation and survival analyses. Third, differences in sequencing platforms and variant annotation protocols across participating institutions could affect consistency in variant reporting and classification. Additionally, the registry currently lacks functional annotations and does not integrate predictive pathogenicity scores or experimental validation, limiting the ability to infer the clinical impact of many variants.

To address these limitations and strengthen the registry’s utility, we hope to be able to implement several improvements in the future, including geographic expansion of data collection to include under-represented and rural populations, standardization of clinical data collection across institutions to improve data quality and comparability, and haplotype analysis to validate suspected founder variants and elucidate population-specific mutation origins. We also hope to be able to integrate automated variant annotation, functional prediction, and classification using tools aligned with ACMG/AMP guidelines. Several of these improvements will require interoperability with national health records databases and regional cancer registries to enable real-time data sharing and clinical integration. However, under the current situation of the Colombian healthcare system, with high fragmentation and very large regional differences in the quality and level of specialization of services offered and the stringent interpretation of privacy regulations, this is not foreseen in the very near future.

Several Latin American countries have published studies on *BRCA1* and *BRCA2* mutations in breast and ovarian cancer patients, offering valuable insights into regional mutation patterns. However, comprehensive national genetic variant registries remain rare in the region. Most efforts are limited to isolated studies without centralized data collection or long-term tracking. This registry represents one of the first coordinated initiatives in Latin America to systematically capture both germline and somatic variants, helping to close a critical gap in cancer genomics and improve regional representation in global databases.

## 5. Conclusions

This study presents the first registry of germline and somatic variants associated with breast and ovarian cancers in Colombia, addressing a critical gap in national genetic data. Among 213 patients, 229 variants were identified, with *BRCA1* and *BRCA2* confirmed as the most frequent germline and somatic mutations. The identification of recurrent and novel variants, including large genomic rearrangements, points to possible founder effects in the Colombian population and underscores the value of targeted genetic screening and the importance of personalized treatment approaches.

This registry contributes not only to the national understanding of cancer genetics but also to global efforts to diversify genomic data sources and reduce disparities in variant interpretation. Despite limitations—including regional bias, heterogeneous clinical data, and absence of functional annotations—the registry establishes a foundational framework for population-specific cancer genomics in Colombia.

Future efforts should focus on expanding the registry’s coverage, improving data standardization, incorporating functional analyses, and fostering collaborations with both national healthcare systems and international variant-sharing platforms. Establishing a permanent, accessible online registry has the potential to significantly improve early diagnosis, guide targeted therapies, and enhance outcomes for patients across Colombia and the broader Latin American region.

## Figures and Tables

**Table 1:** Comparison of age-related epidemiological characteristics among 213 individuals diagnosed with Breast Cancer (BC), Ovarian Cancer (OC), or both. Age at diagnosis is presented as median and interquartile range (IQR). Statistical comparisons were conducted using the Kruskal–Wallis test for continuous variables and Fisher’s exact test for categorical variables. * *p*-values from Kruskal–Wallis (continuous) and Fisher’s exact test (categorical).

Characteristic	Total (n = 213)	BC, n = 93	BC and OC, n = 6		OC, n = 114	*p*-Value *
			BC	OC		
Diagnosis Age (yr, median [IQR])		45 (23–76)	57 (33–82)	59 (41–76)	61 (32–82)	<0.001
Age Group (n; %)						<0.001
20–39		26 (28%)	1 (17%)	0 (0%)	8 (7.0%)	
40–59		58 (62%)	2 (33%)	3 (50%)	75 (66%)	
60–79		9 (10%)	2 (33%)	3 (50%)	30 (26%)	
80–99		0 (0%)	1 (17%)	0 (0%)	1 (1%)	

**Table 2:** Clinical and histopathological characteristics of the 213 individuals with breast and/or ovarian cancer included in the registry. The table summarizes key clinical presentations and tumor subtypes observed in patients diagnosed with Breast Cancer (BC), Ovarian Cancer (OC), or both. For BC cases, histopathological data include the presence of invasive carcinoma, Ductal Carcinoma in Situ (DCIS), and molecular subtypes such as Luminal A, Luminal B, HER2-enriched, and Triple-Negative Breast Cancer (TNBC). For OC cases, histotype are classified into high-grade serous, low-grade serous, endometrioid, clear cell, mucinous, and other subtypes. Instances where data were not reported are indicated as ND (No Data).

Histopathological Characteristics	BC, n = 93	OC, n = 114	BC and OC, n = 6
**Laterality (n; %)**			
Bilateral	10 (11%)		1 (17%)
Right	41 (44%)		4 (66%)
Left	42 (45%)		1 (17%)
**Histologic type (n; %)**			
Invasive	77 (83%)		3 (50%)
Non-invasive (DCIS)	4 (4%)		3 (50%)
ND	12 (13%)		
**Molecular Subtype (n; %)**			
Her-2 Enriched	9 (9.6%)		
Luminal A	14 (15%)		
Luminal B	15 (16%)		2 (33%)
Triple Negative	49 (53%)		
NA	6 (6.4%)		4 (67%)
**Histotype (n; %)**			
Clear cells		2 (1.8%)	
Endometrioid		5 (4.4%)	
Mucinous		1 (0.9%)	
Other		16 (14%)	2 (33%)
High-Grade Serous		87 (76%)	4 (67%)
Low-Grade Serous		3 (2.9%)	

**Table 3:** Distribution of recurrent germline and somatic variants across Colombia’s six geographical regions. This table presents genetic variants observed in three or more individuals (n ≥ 3) within the study cohort, highlighting recurrent mutations of potential clinical and epidemiological relevance. Variants are categorized as germline or somatic and are mapped according to their frequency within the Andean, Caribbean, Pacific, Amazon, Orinoquia, and Insular regions. This analysis provides insight into potential founder effects, regional mutation patterns, and disparities in variant distribution, contributing to a better understanding of population-specific cancer genetics in Colombia.

	Germline Variants							
		Andean	Caribbean	Orinoquia	Insular	Pacific	Amazon	Overall
**Gene**	**HGVS**	**n**	**n**	**n**	**n**	**n**	**n**	**n**
***BRCA1***	c.1674del	2				1		3
	c.3331_3334del	5	1			1		7
	c.5123C>A	4	6	1				11
***BRCA2***	c.2808_2811del	2	1					3
	c.4889C>G	3						3
***Overall***		***16***	***8***	***1***	***0***	***2***	***0***	***27***
	**Somatic Variants**							
***BRCA1***	c.1674del	9						9
	c.3331_3334del	19	1					20
	c.5123C>A	7				1	1	9
***BRCA2***	c.2808_2811del	3				1		4
	c.3860del	4						4
***Overall***		***42***	***1***	***0***	***0***	***2***	***1***	***46***

## Data Availability

Further data and the datasets supporting this study are available from the corresponding author upon justified demand.

## Supplementary Materials

The following supporting information can be downloaded at: https://www.mdpi.com/article/10.3390/diseases13070222/s1, Table S1: Detailed characteristics of the 105 germline variants documented in the national registry. Each variant is described using standardized nomenclature following the guidelines of the Human Genome Variation Society (HGVS). Classification of variants is based on criteria established by the American College of Medical Genetics and Genomics (ACMG), distinguishing pathogenic/likely pathogenic (P/LP) variants from variants of uncertain significance (VUS); Table S2: Detailed characteristics of the 124 somatic variants documented in the national registry. Variant nomenclature follows the standardized guidelines of the Human Genome Variation Society (HGVS). Classification is based on joint criteria established by the American College of Medical Genetics and Genomics (ACMG) and the Association for Molecular Pathology (AMP), distinguishing pathogenic/likely pathogenic (P/LP) variants from variants of uncertain significance (VUS).

## Abbreviations

The following abbreviations are used in this manuscript:

BC	Breast cancer
OC	Ovarian cancer
BOC	Breast and ovarian cancer
P/LP	Pathogenic/likely pathogenic
PARP	poly(ADP-ribose) polymerase
VUS	Variant of uncertain significance
NGS	Next-generation sequencing
REDCap	Research Electronic Data Capture
IQR	Interquartile range
ACMG	American College of Medical Genetics and Genomics
AMP	Association for Molecular Pathology
CC	Collecting center
FFPE	Formalin-fixed paraffin-embedded
LGRs	Large genomic rearrangements
MINAS	Multi-locus inherited neoplasia allele syndrome
TNBC	Triple-negative breast cancer
IRB	Institutional review board
