# Supplementary material for: Establishing the First Genetic Variant Registry for Breast and Ovarian Cancer in Colombia: Insights and Implications
Source: Diseases. 2025 Jul 18;13(7):222. doi: 10.3390/diseases13070222 (PMC12293925; doi:10.3390/diseases13070222)
Supplement: Supplementary file 1 [file diseases-13-00222-s001.zip › diseases-3616972-supplementary.pdf]

Table S1. Detailed characteristics of the 105 germline variants documented in the national registry. Each variant is described using standardized nomenclature following the guidelines of the Human Genome Variation Society (HGVS). Classification of variants is based on criteria established by the American College of Medical Genetics and Genomics (ACMG), distinguishing pathogenic/likely pathogenic (P/LP) variants from variants of uncertain significance (VUS).

HGVS cDNA	HGVS protein effect	ACMG classification	n carriers
	BRCA1		
c.5123C>A	p.Ala1708Glu	Pathogenic	11
c.3331_3334del	p.Gln1111fs	Pathogenic	7
c.1674del	p.Gly559fs	Pathogenic	3
Del Ex14-15	LGR	Pathogenic	3
c.2329T>G	p.Tyr777Asp	VUS	2
c.115T>C	p.Cys39Arg	Pathogenic	1
c.1550A>G	p.Asn517Ser	VUS	1
c.1A>G	p.Met1Val	Pathogenic	1
c.213-12A>G	-	Pathogenic	1
c.3711_3715del	p.Pro1238fs	Pathogenic	1
c.3770_3771del	p.Glu1257fs	Pathogenic	1
c.4163dup	p.Ser1389fs	Likely Pathogenic	1
c.4203del	p.Gln1401fs	Likely Pathogenic	1
c.4327C>T	p.Arg1443*	Pathogenic	1
c.4523G>A	p.Trp1508*	Pathogenic	1
c.5035_5039del	p.Leu1679fs	Pathogenic	1
c.5098del	p.Thr1700fs	Pathogenic	1
c.5159G>A	p.Arg1699Gln	Likely Pathogenic	1
c.5365G>A	p.Ala1789Thr	Pathogenic	1
c.5396-1G>T	-	Pathogenic	1
c.5408G>T	p.Gly1803Val	VUS	1
c.5428G>A	p.Ala1810Thr	Likely Pathogenic	1
c.5518del	p.Asp1840fs	Pathogenic	1
Del Ex17	LGR	Pathogenic	1
	BRCA2		
c.2808_2811del	p.Ala938fs	Pathogenic	3
c.4889C>G	p.Ser1630*	Pathogenic	3
c.3860del	p.Asn1287fs	Pathogenic	2
c.6024dup	p.Gln2009fs	Pathogenic	2
c.6082_6083del	p.Glu2028fs	Pathogenic	2
c.8639_8640del	p.Thr2880fs	Likely Pathogenic	2
c.10095_10096insT	p.Ser3366*	Likely Pathogenic	1
c.171C>A	p.Tyr57*	Pathogenic	1
c.1910-2A>T	-	Pathogenic	1
c.2701del	p.Ala902fs	Pathogenic	1
c.3685G>A	p.Val1229Ile	VUS	1
c.4132dup	p.Thr1378fs	Likely Pathogenic	1
c.5616_5620del	p.Lys1872fs	Likely Pathogenic	1
c.5714A>G	p.His1905Arg	VUS	1
c.5722_5723del	p.Leu1908fs	Pathogenic	1
c.6233dup	p.Val2079fs	Pathogenic	1
c.7426_7428del	p.Glu2476del	VUS	1

c.8169T>A	p.Asp2723Glu	Pathogenic	1
c.8854A>G	p.Met2952Val	VUS	1
c.8953+1G>T	-	Likely Pathogenic	1
c.9259C>A	p.Leu3087Ile	VUS	1
c.9275A>G	p.Tyr3092Cys	VUS	1
c.9648+2dupT	-	Likely Pathogenic	1
	MUTYH		
c.1103G>A	p.Gly368Asp	Pathogenic	2
c.1363A>C	p.Thr455Pro	VUS	1
POLD1			
c.931C>T	p.Arg311Cys	VUS	2
c.631C>T	p.Arg211Cys	Pathogenic	1
APC			
c.7174C>A	p.Pro2392Thr	VUS	1
	ATM		
c.6543G>T	P.Glu2181Asp	VUS	1
c.7187C>G	p.Thr2396Ser	VUS	1
c.7406dup	p.Asn2469fs	Pathogenic	1
C.835A>G	p.Ile29Val	VUS	1
c.8419-2A>C	-	Pathogenic	1
c.8558C>T	p.Thr2853Met	VUS	1
	BRIP1		
c.3390_3393del	p.Tyr1131fs	Pathogenic	1
	CHEK2		
c.1008G>A	p.Gln336=	VUS	1
c.1313A>T	p.Asp438Val	VUS	1
c.1556G>T	p.Arg519Leu	VUS	1
	GALNT12		
c.1A>G	p.Met1?	VUS	1
	MLH1		
c.2271A>T	p.Ter757TyrextTer36	Likely Pathogenic	1
	MSH3		
c.1622C>T	p.Thr541Ile	VUS	1
c.230C>T	p.Pro77Leu	VUS	1
c.2543+4del	-	VUS	1
c.2985G>T	p.Glu995Asp	VUS	1
	NBN		
c.511A>G	p.Ile171Val	VUS	1
	PALB2		
c.1882_1890del	p.Lys628_Cys630del	VUS	1
	PMS2		
c.137G>T	p.Ser46Ile	Pathogenic	1
	POLE		
c.68A>G	p.Ap23Gly	VUS	1
	PTEN		
c.1003C>T	p.Arg335*	Pathogenic	1
	RAD51C		
c.561_562del	p.His187fs	Pathogenic	1

Table S2. Detailed characteristics of the 124 somatic variants documented in the national registry. Variant nomenclature follows the standardized guidelines of the Human Genome Variation Society (HGVS). Classification is based on joint criteria established by the American College of Medical Genetics and Genomics (ACMG) and the Association for Molecular Pathology (AMP), distinguishing pathogenic/likely pathogenic (P/LP) variants from variants of uncertain significance (VUS).

HGVS cDNA	HGVS protein effect	ACMG classification	AMP tier classification	n carriers
		BRCA1		
c.3331_3334del	p.Gln1111fs	Pathogenic	1	20
c.1674del	p.Gly559fs	Pathogenic	1	9
c.5123C>A	p.Ala1708Glu	Pathogenic	1	9
c.5135G>A	p.Trp1712*	Pathogenic	1	2
c.7426_7428del	p.Glu2476del	VUS	NA	2
c.110C>G	p.Thr37Lys	Pathogenic	1	1
c.1252G>T	p.Glu418*	Pathogenic	1	1
c.1293dup	p.Leu432fs	Pathogenic	1	1
c.134+5G>T	-	Likely Pathogenic	1	1
c.1504_1508del	p.Leu502fs	Pathogenic	1	1
c.1999C>T	p.Gln667*	Pathogenic	1	1
c.213-12A>G	-	Pathogenic	1	1
c.2158G>T	p.Glu720*	Pathogenic	1	1
c.226dup	p.Ser76fs	Likely Pathogenic	1	1
c.2278dup	p.Thr760fs	Likely Pathogenic	1	1
c.245T>C	p.Leu82Pro	Likely Pathogenic	1	1
c.2588_2589insA	p.Ser864fs	Likely Pathogenic	1	1
c.2592_2594delinsTTTT	p.Lys865fs	Likely Pathogenic	1	1
c.2702_2703del	p.Phe901fs	Pathogenic	1	1
c.2896_2897del	p.Ile966fs	Pathogenic	1	1
c.2960_2964del	p.Lys987fs	Pathogenic	1	1
c.3270del	p.Gln1090fs	Likely Pathogenic	1	1
c.390C>A	p.Tyr130*	Pathogenic	1	1
c.4097-2A>G	-	Pathogenic	1	1
c.4185G>C	p.Gln1395His	Likely Pathogenic	1	1
c.4249G>A	p.Val1417Met	VUS	3	1
c.4327C>T	p.Arg1443*	Pathogenic	1	1
c.4392del	p.Ile1465fs	Pathogenic	1	1
c.4484+5G>T	-	VUS	1	1
c.4823C>G	p.Ser1608*	Pathogenic	3	1
c.5051del	p.Thr1684fs	Pathogenic	2	1
c.5143A>T	p.Ser1715Cys	Pathogenic	1	1
c.5239A>T	p.Arg1747*	Pathogenic	1	1
c.5329dup	p.Gln1777fs	Likely Pathogenic	1	1
c.5340+1G>T	-	Pathogenic	1	1
c.5342T>A	p.Ile1781Asn	VUS	2	1
c.5396-1G>T	-	Pathogenic	2	1
c.5398C>T	p.Gln1800*	Likely Pathogenic	1	1
c.5428G>A	p.Ala1810Thr	VUS	3	1
c.5574G>A	p.Trp1858*	VUS	2	1
c.607G>T	p.Glu203*	Likely Pathogenic	1	1
c.829_832del	p.Asn277fs	Likely Pathogenic	1	1
Del Ex24	LGR	Pathogenic	-	1
		BRCA2		

c.2808_2811del	p.Ala938fs	Pathogenic	1	4
c.3860del	p.Asn1287fs	Pathogenic	1	4
c.644_646del	p.Glu215del	VUS	2	3
c.10095delinsGAATTATATCT	p.Ser3366fs	Likely Pathogenic	1	1
c.1389_1390del	p.Val464fs	Pathogenic	1	1
c.1411G>T	p.Glu471*	Pathogenic	1	1
c.1528G>T	p.Glu510*	Pathogenic	1	1
c.1763_1766del	p.Asn588fs	Pathogenic	1	1
c.2050C>T	p.Gln684*	Likely Pathogenic	1	1
c.2068G>T	p.Glu690*	Likely Pathogenic	1	1
c.2979G>A	p.Trp993*	Pathogenic	1	1
c.3187C>T	p.Gln1063*	Pathogenic	1	1
c.3994C>G	p.His1332Asp	VUS	3	1
c.4889C>G	p.Ser1630*	Pathogenic	1	1
c.4936_4939del	p.Glu1646fs	Pathogenic	1	1
c.5054C>T	p.Ser1685Leu	VUS	3	1
c.5073dup	p.Trp1692fs	Pathogenic	1	1
c.5073dupA	p.Trp1692fs	Pathogenic	1	1
c.5557dup	p.Cys1853fs	Pathogenic	1	1
c.5851_5854del	p.Ser1951fs	Pathogenic	1	1
c.6024dup	p.Gln2009fs	Pathogenic	1	1
c.6250G>A	p.Asp2084Asn	VUS	3	1
c.6884_6888del	p.Arg2295fs	Pathogenic	1	1
c.7006C>T	p.Arg2336Cys	VUS	3	1
c.7007G>A	p.Arg2336His	Pathogenic	1	1
c.7063G>T	p.Glu2355*	Pathogenic	1	1
c.7436-1G>A	-	Pathogenic	1	1
c.7674_7676del	p.Glu2558_Ser2559delinsAsp	VUS	2	1
c.8009C>T	p.Ser2670Leu	Pathogenic	1	1
c.8247_8248del	p.Lys2750fs	Pathogenic	1	1
c.8487_8487+2delGGTinsATA	-	Likely Pathogenic	1	1
c.8699A>T	p.Asp2900Val	VUS	3	1
c.8953+2T>A	-	Likely Pathogenic	1	1
c.9004G>A	p.Glu3002Lys	Pathogenic	1	1
c.994del	p.Ile332fs	Pathogenic	1	1
		PALB2		
c.2288_2291del	p.Leu763fs	Pathogenic	1	1
